# Quality assurance evaluation of delivery of respiratory‐gated treatments

**DOI:** 10.1120/jacmp.v5i3.2002

**Published:** 2004-10-21

**Authors:** Alex Cardenas, Jonas Fontenot, Kenneth M. Forster, Craig W. Stevens, George Starkschall

**Affiliations:** ^1^ Departments of Radiation Physics The University of Texas M. D. Anderson Cancer Center 1515 Holcombe Boulevard Houston Texas U.S.A. 77030; ^2^ Radiation Oncology The University of Texas M. D. Anderson Cancer Center 1515 Holcombe Boulevard Houston Texas U.S.A. 77030

**Keywords:** respiratory gating, quality assurance

## Abstract

We describe a method for evaluating the quality of respiratory‐gated radiation delivery using a commercially available device. During irradiation, gating traces for one field for each treatment were extracted from the system for each of 14 patients. The data were then transferred to a spreadsheet. Software was developed to evaluate the following parameters: duty cycle, amplitude of fiducial motion, fraction of amplitude of motion during gated delivery, and respiratory cycle time. Criteria were established for acceptability of gating traces. In our sample, over 85% of the traces indicated acceptability. An example of results for one patient extracted from analyzed gating traces is as follows: mean duty cycle, 57%; average amplitude of motion, 0.89 cm; average fraction of motion during gated delivery, 0.45; mean respiratory cycle time, 4.5 s. This technique can be used to evaluate delivery of respiratory‐gated radiation therapy for quality assurance purposes and to assess various techniques for improving delivery of gated therapy. A hard copy of the gating traces can be used to document gated treatment delivery for potential billing of the gated delivery process.

PACS numbers: 87.53‐j, 87.53.Tf

## I. INTRODUCTION

During preradiation treatment planning, adequate margins must be established around the clinical target volume (CTV) to account for tumor motion. The size of the margins must be determined in consideration of two treatment goals: complete irradiation of the CTV despite its motion and the need to minimize the amount of uninvolved tissue irradiated as a result of the larger treatment portals necessary to irradiate the target plus margin. The need to minimize the margins for motion is especially important in the case of respiratory‐induced lung tumor motion because of the relatively low radiation doses needed to damage lung tissue. Gated delivery^(^ ^1^ ^,^ ^2^ ^)^ has been used as a method for reducing the size of the margin needed to account for respiratory motion. This method consists of turning the radiation beam on and off based on the motion of the tumor or a tumor surrogate. As a consequence of the beam‐off periods, the delivery of radiation will take longer than it would for ungated treatment delivery.

A respiratory gating system is used to monitor respiratory motion by tracking an external fiducial using an infrared (IR) light source and a charge‐coupled device (CCD) detector. This system triggers delivery of radiation at a specified point in the respiratory cycle on the basis of either displacement of the fiducial or the phase of the respiratory cycle as established by a predictive filter. Previous work with respiratory gating has been reported in the literature.^(^ ^3^ ^–^ ^5^ ^)^

In the radiation oncology clinic, it would be useful to monitor the effectiveness of respiratory gating. Some parameters that could be monitored are the fraction of time the beam is on, the amplitude of fiducial displacement, the fraction of this amplitude that occurs during radiation delivery, and the mean respiratory cycle time. These quantities are important, because in order to deliver the prescribed dose to the patient, the therapist may need to account for the beam‐off periods and adjust the maximum permitted time the beam is on. An estimate of the quality of the gating process might be inferred from an estimate of the magnitude of residual motion while the radiation beam is on. Finally, it would be useful to have a hard copy record of the gating session as documentation of the session and to provide a record that could be used for potential billing purposes. The objectives in this study were to develop a procedure for tracking and recording residual motion and duty cycle, and to generate a hard copy record of the gating process.

## II. MATERIALS AND METHODS

### A. Respiratory gating system

**Figure 1:**
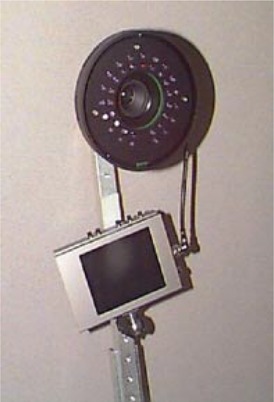
The IR source and CCD detector that constitute the Varian RPM™ system

The commercially available respiratory gating system (RPM™, Varian Medical Systems, Palo Alto, CA) consists of a marker block, an IR light source, a CCD tracking camera, a viewfinder used to visualize the relative position of the block, and a workstation that displays and records the motion data.^(6)^ The marker block consists of two reflective fiducials that are placed 3 cm apart on the patient's abdomen. Abdominal motion is used as a surrogate for respiratory‐induced tumor motion. The system monitors abdominal motion by tracking the external fiducials using the IR light source and CCD detector. The respiratory gating system is installed on three LINACs (Clinac 21EX, Varian Medical Systems) in our clinic (Fig. 1).

### B. Patient selection

Fourteen patients diagnosed with a variety of tumors and treated with respiratory‐gated delivery were selected for this study. Patients were selected because of tumors in the thorax or abdomen where respiratory‐induced motion may be significant. Table 1 summarizes the patients in the study.

**Table 1:** Summary of patients included in study

Patient	Tumor location	Stage	Diagnosis
1	left bronchus/lung	I	adenocarcinoma
2	liver	III	adenocarcinoma
3	liver	unresectable	intrahepatic cholangiocarcinoma
4	left upper lobe	I	nonsmall cell
5	right middle lobe	IV	nonsmall cell
6	right upper lobe	IIIB	nonsmall cell
7	right upper lobe	III	nonsmall cell
8	right lower lobe	II	adenocarcinoma
9	right upper lobe	I	nonsmall cell
10	left lung	III	squamous cell carcinoma
11	liver	IV	adenocarcinoma
12	left upper lobe	I	nonsmall cell
13	right upper lobe	I	nonsmall cell
14	left upper lobe	II	nonsmall cell

### C. Patient monitoring

Despite evidence that more normal lung tissue is spared at deep inspiration,^(7)^ our clinical practice is to gate at end expiration using amplitude‐based gating. We have found end‐expiration gating to be more reproducible and a more comfortable breathing position for the patient.^(7)^ Treatment planning was done using computed tomography (CT) image data sets acquired by triggering a helical CT scanner (PQ‐5000, Philips Medical Systems, Cleveland, OH) operating in axial mode to acquire single axial scans at end expiration.

At the time of treatment, the reflective box was placed in the patient's abdomen in the same location as it was placed for simulation. All radiation fields were delivered under gating, but only the gating record from the last field treated each day was stored. Records for each of the 14 patients were acquired for the duration of their treatments.

### D. Data analysis

The raw gating data were transferred from the gating computer to a spreadsheet (Excel, Microsoft Inc., Redmond, WA). The spreadsheet contained a macro that was written to analyze the data. The macro first extracted the time elapsed since the beginning of the beam on, the vertical location of the reflective box, and a flag that indicated whether or not the beam was on. Once these values were known, the macro then calculated the respiratory cycle time, the amplitude of motion (total vertical displacement), the duty cycle (ratio of beam‐on time to total treatment time), and the fraction of amplitude of motion occurring when the beam was on. These calculations were based on the fact that every data point is acquired at a regular time interval. Therefore, between any two consecutive readings, the time elapsed was the same. For instance, when the macro computed the duty cycle, it counted how many data points were flagged as “beam on” and divided that number by the total number of data points. This ratio was then converted to a percentage. To obtain the respiratory cycle time, the total time was divided by the number of breathing cycles. Total time was computed by multiplying the number of data points times the duration between any two data points.

The number of breathing cycles was obtained from the gating record by counting the number of instances when the beam went from on to off. To obtain the amplitude of motion, the macro searched for the maximum and for the minimum displacement in each cycle, and then computed the difference between the displacements. Finally, to determine the fraction of amplitude of motion while the beam was on, the macro identified the data point at which the beam goes from on to off. The difference between the displacement at this data point and the minimum displacement was divided by the amplitude of motion for that particular cycle.

## III. RESULTS

**Figure 2:**
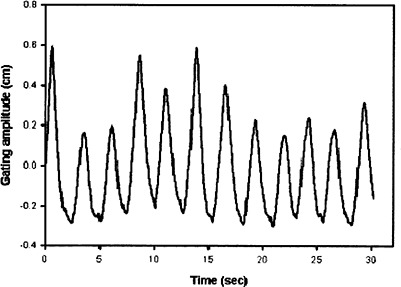
Amplitude of vertical motion of reflective box during a patient treatment

**Figure 3:**
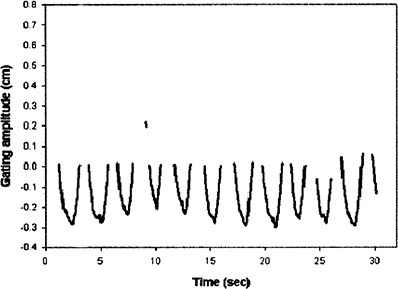
Amplitude of motion while radiation was being delivered under gating

Figures 2 and 3 illustrate examples of the plots of a gating trace. Figure 2 illustrates the complete gating trace for a single treatment, while Fig. 3 illustrates the gating trace only for the time period that radiation was delivered.

For a typical patient, 85% of traces indicated satisfactory delivery of radiation. The remaining 15% did not necessarily indicate a problem in delivering the radiation, but rather that in some cases, the gating traces did not record the proper signal. Further investigation with the vendor led to identification of a software problem that caused the error in recording the gating signal, which was subsequently corrected. The results of the gating analysis for each of the 14 patients are presented in Table 2. The tabulated numbers represent mean values and standard deviations.

**Table 2:** Results of gating analysis for each patient

Patient	Duty cycle	Respiratory amplitude	Fraction of motion during delivery	Respiratory cycle time
1	56.7±14.9%	0.89±0.26 cm	0.45±0.14	4.55±1.47 s
2	69.2±12.3%	0.66±0.22 cm	0.53±0.13	3.60±0.78 s
3	46.1±13.0%	0.97±0.33 cm	0.44±0.18	3.07±0.39 s
4	40.0±24.0%	0.74±0.55 cm	0.47±0.27	2.71±1.25 s
5	34.3±14.1%	0.95±0.26 cm	0.35±0.14	4.74±1.84 s
6	48.3±23.0%	0.84±0.60 cm	0.43±0.22	3.55±1.16 s
7	42.0±16.1%	1.17±0.64 cm	0.33±0.15	6.27±4.37 s
8	41.3±12.0%	0.83±0.34 cm	0.36±0.11	4.41±1.39 s
9	47.2±16.4%	0.89±0.38 cm	0.36±0.15	3.21±0.76 s
10	60.7±14.0%	0.98±0.55 cm	0.57±0.21	4.41±1.51 s
11	48.0±11.7%	1.67±1.03 cm	0.37±0.21	6.76±1.45 s
12	45.3±18.0%	1.04±0.32 cm	0.42±0.21	4.59±1.57 s
13	67.9±14.0%	0.77±0.35 cm	0.58±0.18	2.96±1.33 s
14	29.0±3.8%	1.55±0.16 cm	0.16±0.07	9.8±0.52 s

The duty cycle varied among patients from 29.0% to 69.2%, resulting in an increase in delivery times by a factor that ranged from 1.44 to 3.45. There was also significant variation per patient in length of treatment time on different days, as reflected in the standard deviation of the duty cycle, which we observed to be as great as 24%. The same variations were noticeable in the respiratory amplitude. This quantity, which ranged from 0.66 cm to 1.67 cm, is actually the vertical displacement of the marker box on the patient's surface. A 1.1‐cm difference may seem small, but these numbers indicate that the movement of the marker can be up to 2.5 times as large for one patient as compared to another, suggesting a wide variation in magnitude of respiratory‐induced motion. Also, for the same patient, the amplitude of motion varied as much as 1.03 cm. This variation indicates the need for accurate tracking of the motion of the tumor for delivery of radiation. The fraction of amplitude of motion during radiation delivery varied from 0.16 to 0.58, indicating that for some patients, tumor motion during gated treatment was relatively small, whereas for other patients, tumor motion remained significant.

**Figure 4:**
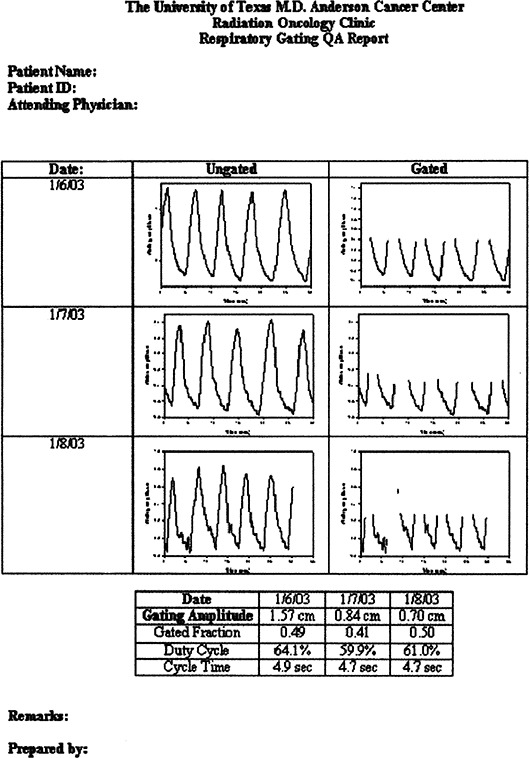
Sample recording form for gated treatment delivery. The form displays three gated treatments, although typically perhaps 30 treatments might be displayed.

We also developed recording forms that can be used to summarize the gated delivery of radiation (Fig. 4). These forms can be included with the patient's treatment record to verify that gated treatment was actually delivered and can serve as a record for billing.

## IV. DISCUSSION AND CONCLUSIONS

Using a simple macro on a commercially available spreadsheet, we can record and analyze data associated with gated treatment delivery. The spreadsheet allows us to track and record residual motion and duty cycle for each patient, as well as other parameters. The values of these parameters can vary greatly from patient to patient, and, indeed, from day to day for the same person, as observed by the standard deviations recorded for our study group. These variations are usually caused by different factors, such as lung vital capacity, tumor size, stage of disease, and even the patient's emotional state. For example, a patient might have more difficulty breathing during earlier treatment sessions than during later sessions when the tumor might be smaller due to radiation. A patient may also breathe heavily and shallowly during the first few treatment days when he or she is more anxious and then calmly and deeply later once the patient is more comfortable with the procedure.

The information regarding all these parameters could be used by the physician or therapist during treatment. However, we must take into account that, although the gating device tracks one‐dimensional motion, the tumor itself moves in three dimensions. This factor might increase the uncertainty of the motion error. The range of variations observed in this study indicates the need for a recording system. The objective of this project has been the creation of such a recording system. Review of gating traces allows the therapist to modify gating thresholds to determine the appropriate magnitude of residual motion and duty cycle to use during radiation therapy. This information is also useful as a quality assurance tool for improving the quality of gated delivery. For example, such data could be used to evaluate the trade‐offs and make better decisions regarding the reduction of displacement under gated delivery versus the increase in duty cycle.

## ACKNOWLEDGMENT

This research was supported in part by a sponsored research agreement with Varian Medical Systems, Inc., Palo Alto, CA.
